# Liquid crystal delivery of ciprofloxacin to treat infections of the female reproductive tract

**DOI:** 10.1007/s10544-019-0385-x

**Published:** 2019-03-29

**Authors:** Simone Pisano, Matteo Giustiniani, Lewis Francis, Deyarina Gonzalez, Lavinia Margarit, I. Martin Sheldon, Donatella Paolino, Massimo Fresta, R. Steven Conlan, Gareth D. Healey

**Affiliations:** 1 Institute of Life Science, Swansea University Medical School, Swansea University, Swansea, SA2 8PP UK; 2 Department of Clinical and Experimental Medicine, University of Catanzaro “Magna Graecia”, Viale “S. Venuta”, 88100 Catanzaro, Italy; 3 Inter-Regional Research Center for Food Safety & Health, University of Catanzaro “Magna Græcia”, Viale “S. Venuta”, 88100 Catanzaro, Italy; 4 Obstetrics & Gynecology Department Princess of Wales Hospital, Abertawe Bro Morannwg University Health Board, Coity Road, Bridgend, CF31 1RQ UK

**Keywords:** Liquid crystal, Drug delivery, Female reproductive tract, Bacterial infection, Female reproductive tract infection, Ciprofloxacin

## Abstract

Infections of the female reproductive tract are a major cause of morbidity and mortality in humans, requiring significant investment to sustain treatment and representing a major challenge to health. The increasing prevalence of bacterial resistance, and an almost complete absence of new antibiotic therapies for the past five decades, mean there is a desperate need for novel approaches to the treatment of bacterial infections. Within the present study, we demonstrate the effective *ex vivo* treatment of bacterial infection of the female reproductive tract using a controlled-release, liquid crystal-based platform. Liquid crystal encapsulation of ciprofloxacin significantly enhanced its bactericidal efficacy and reduced cell toxicity. Liquid crystal structures are low-cost, simple to manufacture and provide a sustained-release profile of encapsulated ciprofloxacin. Treatment of *Escherichia coli* infected reproductive tract epithelial cells and whole organ cultures with liquid crystal encapsulated ciprofloxacin proved to be an effective strategy for reducing bacterial load and reproductive tract inflammatory responses to infection. These data suggest that such an approach could provide an efficacious treatment modality for enhancing the effectiveness of current antibiotic therapies.

**Electronic supplementary material:**

The online version of this article (10.1007/s10544-019-0385-x) contains supplementary material, which is available to authorized users.

## Introduction

Sexually transmitted infections and their associated diseases represent one of the main challenges in worldwide reproductive health (Horne et al. 2008). Despite sustained activity aimed at preventing such infections, they remain a significant socio-economic burden with the most prevalent causative agents being Herpes simplex virus type 2, group B streptococcus, *Treponema pallidum* (syphilis), bacterial vaginosis, Hepatitis B virus (hepatitis), *Neisseria gonorrhoeae* (gonorrhea), *Chlamydia trachomatis*, and human immunodeficiency viruses (HIVs) (Wira et al. 2011). According to World Health Organization (WHO) estimates, in 2008 there were more than 498 million new cases of over 30 different sexually transmitted infections. These included infection with *Trichomonas vaginalis* (276 million new cases), *Chlamydia trachomatis* (106 million new cases)*, Treponema pallidum* (10 million new cases), HIV (2.7 million new cases) and *Neisseria gonorrhoeae* (106 million new cases) (WHO 2012).

The female reproductive tract has an abundant natural bacterial flora, dominated in most women by *Lactobacillus* species, which represent 90–95% of the total bacteria in the reproductive tract (Delaney and Onderdonk 2001; Larsen and Monif 2001; Srinivasan et al. 2010). *Lactobacillus*-dominance is not universal however, with several studies showing the presence of a diverse, heterogeneous microbiota that extends throughout the reproductive tract in women without reproductive tract disease (Chen et al. 2017; Lambert et al. 2013; Ravel et al. 2013; Srinivasan et al. 2010). *Lactobacillus* species also do not dominate throughout a woman’s lifetime, with anaerobes and *Escherichia coli* most abundant during childhood and after menopause, when lower estrogen levels reduce the abundance of glycogen in the vaginal epithelium, which is essential for *Lactobacillus* growth and colonization (Brotman et al. 2014; Galhardo et al. 2006; Hillier and Lau 1997). The presence of *Lactobacillus* species is currently accepted as a biomarker of healthy status (Amabebe and Anumba 2018; das Neves et al. 2014). Through the production of lactic acid, which lowers vaginal pH, together with the production of hydrogen peroxide, bacteriocins and other antimicrobial compounds, *Lactobacilli* help to maintain vaginal homeostasis by creating an environment unsuitable for neutrophilic bacteria and by competing for nutrients, which deters the growth of pathogenic bacteria (Barbés and Boris 1999; Eschenbach et al. 1989; Tachedjian et al. 2018). Indeed, modification of these physiological conditions to a pH above 4.5 increases the risk of other bacterial infections including sexually transmitted infections and those associated with bacterial vaginosis (Aldunate et al. 2015; Daniel Johannes Rönnqvist et al. 2006; Mastromarino et al. 2014; McLaughlin et al. 2018).

Early and effective treatment of reproductive tract infections is essential to limit transmission of disease and to reduce and/or prevent associated morbidities. The development of antibiotic resistance by pathogens to many antibiotics has increased concerns about the effective management of these diseases (Alirol et al. 2017; Bala et al. 2007; P. Health England 2013; Laibl et al. 2005; Martin et al. 2006; McCarthy 2007; MMWR 2006; Nys et al. 2004; Wang et al. 2005). For example, a positive correlation between ciprofloxacin resistance and gonorrhea incident rates has been reported in several countries leading to the drug being removed as the primary treatment option (Bala et al. 2007; Chesson et al. 2014; McCarthy 2007; MMWR 2006). Therefore, finding a way to optimally identify patients more likely to respond positively to an antibiotic regimen can represent a competitive advantage for disease management. With this in mind, the UCLA Health Group introduced a genotypic assay to predict the susceptibility of *N. gonorrhoea* to ciprofloxacin, thus repositioning this drug as a valuable tool if the correct patient cohort is identified (Allan-Blitz et al. 2016). The same approach is true for other bacterial infections. In addition, a drive to reduce the amount of administered drug, which favors a more localized treatment approach, would reduce negative side effects such as diarrhea, nausea and vomiting, and possibly delay the onset of drug resistance.

Localised treatments of reproductive tract infections are favoured over oral approaches because they achieve higher local drug concentrations and avoid affecting the gastrointestinal tract microbiome (Alexander et al. 2004; Vermani and Garg 2000). Several different delivery modalities are currently marketed including tablets, capsules, pessaries and semisolids (Henderson 1958; Das Neves and Bahia 2006; Pavelić et al. 2005; Slack and Nichols 1982; Yao et al. 2016), but most are associated with poor distribution and retention due to the self-cleaning action of the vagina (Hussain and Ahsan 2005). A drive towards the use of semisolids (creams or gels), due to the ease of application and the symptomatic relief provided by the additional moisture provided, is evident (das Neves 2014; Palmeira-de-Oliveira et al. 2015).

The need to improve drug delivery beyond the classical pharmaceutical forms has driven the development of novel delivery systems designed to improve patient compliance, enhance cellular targeting, extend drug release profiles and reduce harmful side-effects. Several of these new pharmaceutical compositions such as liposomes, polymeric systems or cyclodextrins, are already marketed. Their advantage is to offer improved therapeutic profiles by encapsulating a drug, thereby protecting it from degradation, whilst also providing the opportunity for the attachment of targeting molecules on the outside of the carrier (Chen et al. 2014).

Liquid crystals are an emerging class of drug delivery systems made of polar lipids able to spontaneously reorganize themselves into three-dimensional structures (namely liquid crystalline phases) when in contact with water (Shah et al. 2001). They have a malleable structure, determined by the local physico-chemical properties, meaning their structure can be controlled to allow both ease of administration and sustained drug release (Chaudhary et al. 2016). Liquid crystals exhibit both optical and electrical anisotropy, as well as flow properties and molecular mobility. Whilst in practice liquid crystals do not transition directly from a liquid to a solid state, under certain conditions they exhibit molecularly organised intermediate phases (mesophases) with liquid and solid state properties (Zabara and Mezzenga 2014). Liquid crystals that form mesophases in appropriate solvents, named lyotropic liquid crystals, are based on lipids that spontaneously self-assemble in an aqueous environment. They are characterised by nanostructured hydrophilic and hydrophobic domains separated by lipid bilayers, and form following exposure to a polar (aqueous) environment (Zabara and Mezzenga 2012).

In the present study we evaluate controlled antibiotic delivery in cell and tissue models using ciprofloxacin, a second-generation fluoroquinolone, with broad-spectrum antibiotic activity against both Gram-positive and Gram-negative bacteria (LeBel 1988; Wiseman and Balfour 1994) encapsulated in liquid crystal. By demonstrating how liquid crystals can be used as a drug delivery system in the treatment of bacterial infections of the female reproductive tract, and that liquid crystal formulations of ciprofloxacin significantly enhance bacterial killing compared to the free drug formulation, we pave the way for tailored, controlled-release treatments of bacterial infections within the female reproductive tract.

## Methods and materials

### Preparation of lamellar phase ciprofloxacin emulsion (95% Monomuls® + 5% - 0.05% TFA)

A solution of 0.05% trifluoroacetic acid (TFA, Sigma-Aldrich, Dorset, UK) was prepared in deionised water, and ciprofloxacin (Sigma-Aldrich) was dissolved in it to the desired final concentration. The monoemulsion solution was obtained by adding 4.75 g of Monomuls® 90–018 (BASF, Cheadle, UK) to a glass beaker and heating it to 40 °C in a water bath. Once the monoemulsion became molten, 250 μl of the ciprofloxacin solution were added and the emulsion mixed. The ciprofloxacin emulsion could be maintained in the lamellar phase at 37 °C until needed.

### Preparation of cubic phase ciprofloxacin emulsion (75% Monomuls® + 25% - 0.05% TFA)

A solution of 0.05% TFA was prepared in deionised water, and ciprofloxacin was dissolved in it to the desired final concentration. The monoemulsion solution was obtained by adding 3.25 g of Monomuls® 90–018 into a glass beaker and heating it to 40 °C in a water bath. Once the monoemulsion became molten, 1250 μl of the ciprofloxacin solution was added and mixed with a glass wand. The solution was cooled to ambient room temperature, undergoing a phase shift to the cubic structure in the process, and stored at room temperature until needed.

### Characterization of liquid and cubic phases by scanning electron microscopy

Analysis was performed as previously described (Tan et al. 2008). Briefly, samples were deposited on a silicon surface and snap frozen in liquid nitrogen to ensure the conformational stability of the liquid crystals. The product was then coated with a 2 nm thick carbon coating and SEM images were acquired using an S-4800 scanning electron microscope (Hitachi) operated at accelerating voltages of 2 or 10 kV.

### Drug release profiling by high-pressure liquid chromatography (HPLC)

The release of ciprofloxacin from cubic phase ciprofloxacin emulsion was measured using dialysis membrane and HPLC. Cubic phase ciprofloxacin emulsion was placed into dialysis tubing cellulose membrane (14,000 MWCO, Sigma-Aldrich, UK) and each end of the tubing sealed. The sealed membrane was then placed in a solution of 0.05% TFA in deionised water and samples extracted at various time points over a 72-h period and stored at 4 °C ready for HPLC analysis. HPLC analysis was performed as previously described by Locatelli and colleagues (Locatelli et al. 2015).

### Primary cell and organ culture

Uteri with no gross evidence of reproductive disease or microbial infection, were collected from post-pubertal mixed-breed beef cattle (*n* = 16 over a 3-month period) within 15 min of slaughter as part of the routine operation of a commercial slaughterhouse. Postpartum cattle were not used because of the ubiquitous bacterial contamination and disruption of the epithelium that is typical of the puerperal endometrium (Herath et al. 2009; Wathes et al. 2009). The animals were 20–26 months old, reared on extensive grassland and had never been pregnant or inseminated. The stage of reproductive cycle was determined by examination of ovarian morphology and vasculature, as described previously, and animals on days 1 to 4 of the oestrus cycle were used because, similar to postpartum cows, peripheral plasma ovarian hormone concentrations are basal (Ireland et al. 1979). The uteri were kept on ice for approximately 1 h until further processing at the laboratory. External surfaces were washed with 70% ethanol and the uterine horn opened longitudinally with sterile scissors. Since innate immune responses are the same irrespective of the horn used, one horn was used for the isolation of purified endometrial cell populations, and the contralateral horn used for organ culture (Saut et al. 2014).

Endometrial cells were isolated as described previously (Cronin et al. 2012; Herath et al. 2006). Epithelial and stromal cell populations were distinguished by cell morphology, the presence of cytokeratin and vimentin respectively, and the absence of immune cell contamination was confirmed by the absence of CD45, as described previously (Herath et al. 2006; Turner et al. 2014). The epithelial and stromal cells were cultured in 1 ml complete medium per well, comprising: Phenol-red free Roswell Park Memorial Institute (RPMI) 1640 medium (Sigma-Aldrich, Dorset, UK) containing 10% heat-inactivated foetal bovine serum (FBS; Biosera, East Sussex, UK), and plated at 1 × 10^5^ cells/ml in 24-well plates (TPP, Trasadingen, Switzerland) ready for treatment. *Ex vivo* organ cultures (EVOC) of the endometrium were collected using 8 mm diameter punch biopsies as previously described (Borges et al. 2012). Tissues were cultured in 24-well plates (TPP) containing 2 ml complete medium per well, and treatments initiated within 4 h of slaughter. During treatment, cells or tissues were maintained in a humidified, 5% CO_2_ in air atmosphere incubator at 37 °C, with supernatants collected as indicated.

### Immortalised cell-lines culture

The human HeLa cervical epithelial and human HEC1A endometrial epithelial cell lines were obtained from the European Collection of Cell Cultures (ECACC, Salisbury, UK) and cultured in Dulbecco’s Modified Eagle Medium/F-12 nutrient mix (Thermofisher, Gloucester, UK) supplemented with 10% foetal bovine serum (FBS). Sub-confluent cultures were split using trypsin/ethylenediaminetetraacetic acid (EDTA) and seeded into 24-well plates (TPP, Trasadingen, Switzerland) in 1 ml of medium at 1 × 10^5^ cells/ml for bacterial infection experiments. Or were seeded onto 0.4 μm collagen-coated Transwell™ support membranes (Sigma-Aldrich, Dorset, UK) in 0.25 ml of medium at 1 × 10^5^ cells/ml for cell viability experiments.

### Cell viability

Cell viability was assessed by the mitochondria-dependent reduction of MTT to formazan, as described previously (Mosmann 1983). The correlation between MTT OD_570_ measurements and the number of live cells was confirmed using trypan blue exclusion and counting the number of live cells using a haemocytometer.

### Bacterial culture

Cultures of *E. coli* (isolate MS499), collected from the uteri of postpartum cows with persistent uterine disease, were grown overnight in Luria-Bertani (LB) medium (Sigma-Aldrich) as described previously (Amos et al. 2014; Sheldon et al. 2010). The bacteria were diluted to 1 × 10^8^ colony forming units (CFU)/mL in medium and centrifuged at 1500×g for 10 min at room temperature. The bacteria were then resuspended in sterile PBS (Life Technologies Ltd., Paisley, UK) followed by centrifugation at 6000×g for 10 min at 4 °C. After washing, bacteria were diluted to 1 × 10^3^ CFU/ml in LB medium for bactericidal experiments, or complete cell culture medium for cell/organ infection experiments. Where required, the number of colony-forming units (CFU) was determined by plate counts on LB agar plates following serial dilution of bacterial cultures and/or cell/organ culture supernatants.

### Enzyme-linked immuno sorbent assay (ELISA)

Concentrations IL-6 in cell and EVOC culture supernatants were measured by ELISA according to the manufacturer’s instructions Bovine IL-6 Screening Set ESS0029, ThermoFisher Scientific). To account for differences between the weights of EVOC tissues, concentrations are reported as pg per mg tissue. The limit of detection for IL-6 was 35.6 pg/ml; the intra-assay coefficient of variance was 1.2% and the inter-assay coefficient of variance was 3.0%.

### Statistical analysis

Statistical analyses were performed using IBM SPSS Statistics 20. Initially the data were tested for homogeneity, and log or square root transformed if appropriate. Data were analysed by analysis of variance (ANOVA) using Dunnett’s pairwise multiple comparison t-test for individual group comparisons, or by student’s t test. Data are presented as mean with standard deviation (SD), *P* < 0.05 was considered statistically significant, and n represents the number of independent experiments.

## Results

### The preparation of liquid crystal emulsions is simple and reproducible

**Fig. 1:**
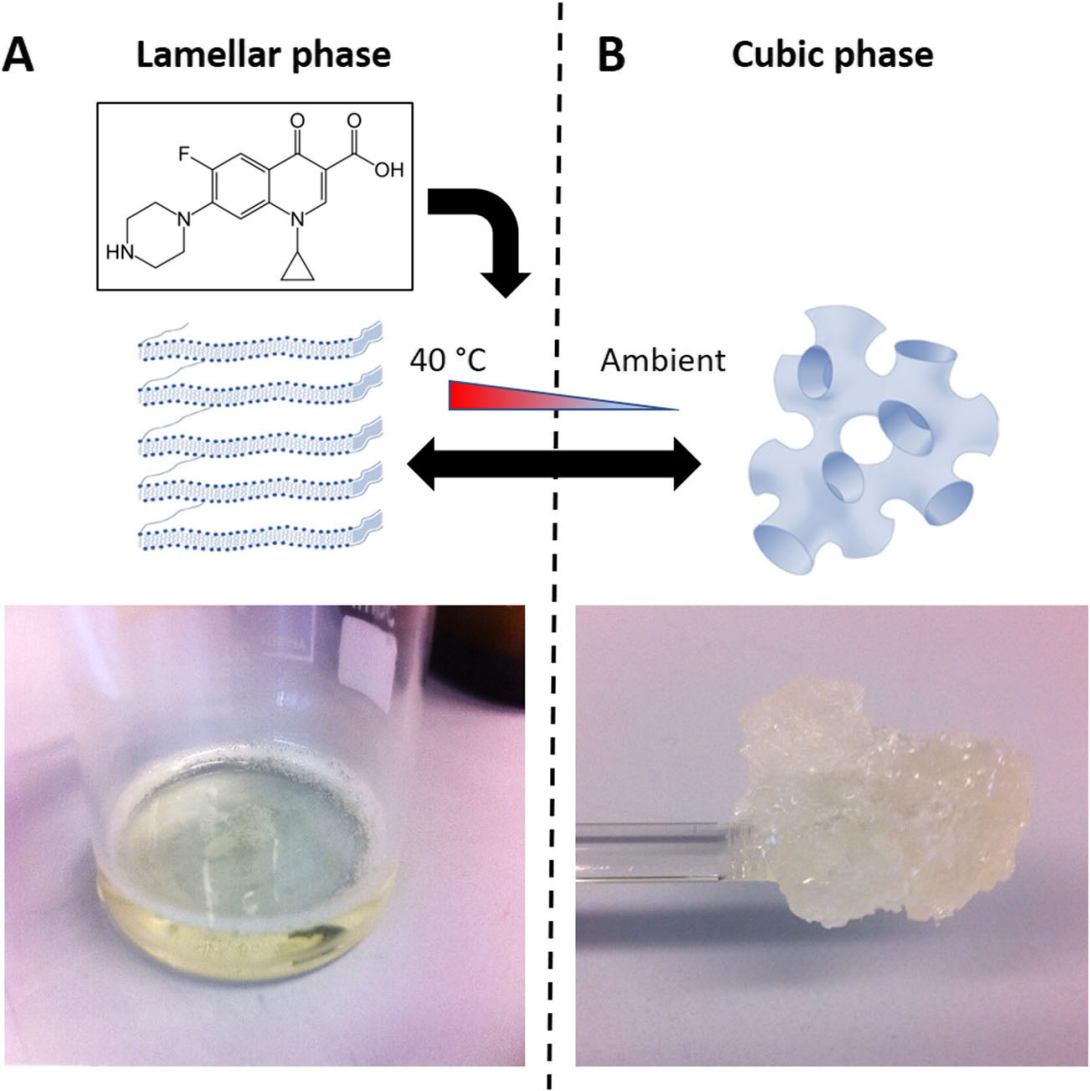
Liquid crystal emulsions were prepared in different phases through controlled application of water content and heat: **a** Lamellar phase emulsions were obtained by mixing Monomuls® with a solution of 0.05% TFA (95% to 5%, *w*/*v*), heating the solution to 40 °C and then maintaining at 37 °C; **b** Cubic phase emulsions were obtained by mixing Monomuls® with a solution of 0.05% TFA (75% to 25%, *w/v*) and after heating to 40 °C, the solution was cooled to ambient room temperature, undergoing a phase shift to the cubic structure in the process, and stored at room temperature until needed. Ciprofloxacin was incorporated into the liquid crystal structures by addition whilst at 40 °C

The preparation of different liquid crystal encapsulated ciprofloxacin emulsions was achieved by altering the proportion of water used during synthesis (Fig. 1). Lamellar structures (Fig. 1a) were prepared using a lower proportion of water (5% compared to 25% in cubic phase preparations) and maintained in the lamellar phase by heating to 40 °C. Cubic structures (Fig. 1b) were maintained at ambient room temperature and readily phase shifted between a cubic and lamellar structure by the application of heat (40 °C). For both structures, ciprofloxacin was added whilst in the lamellar phase and becomes incorporated within the crystal structure once it is heated to 40 °C. Drug equivalencies were calculated based on the total amount of ciprofloxacin added and the total volume of the emulsion whilst the liquid crystal was in the lamellar phase.

**Fig. 2:**
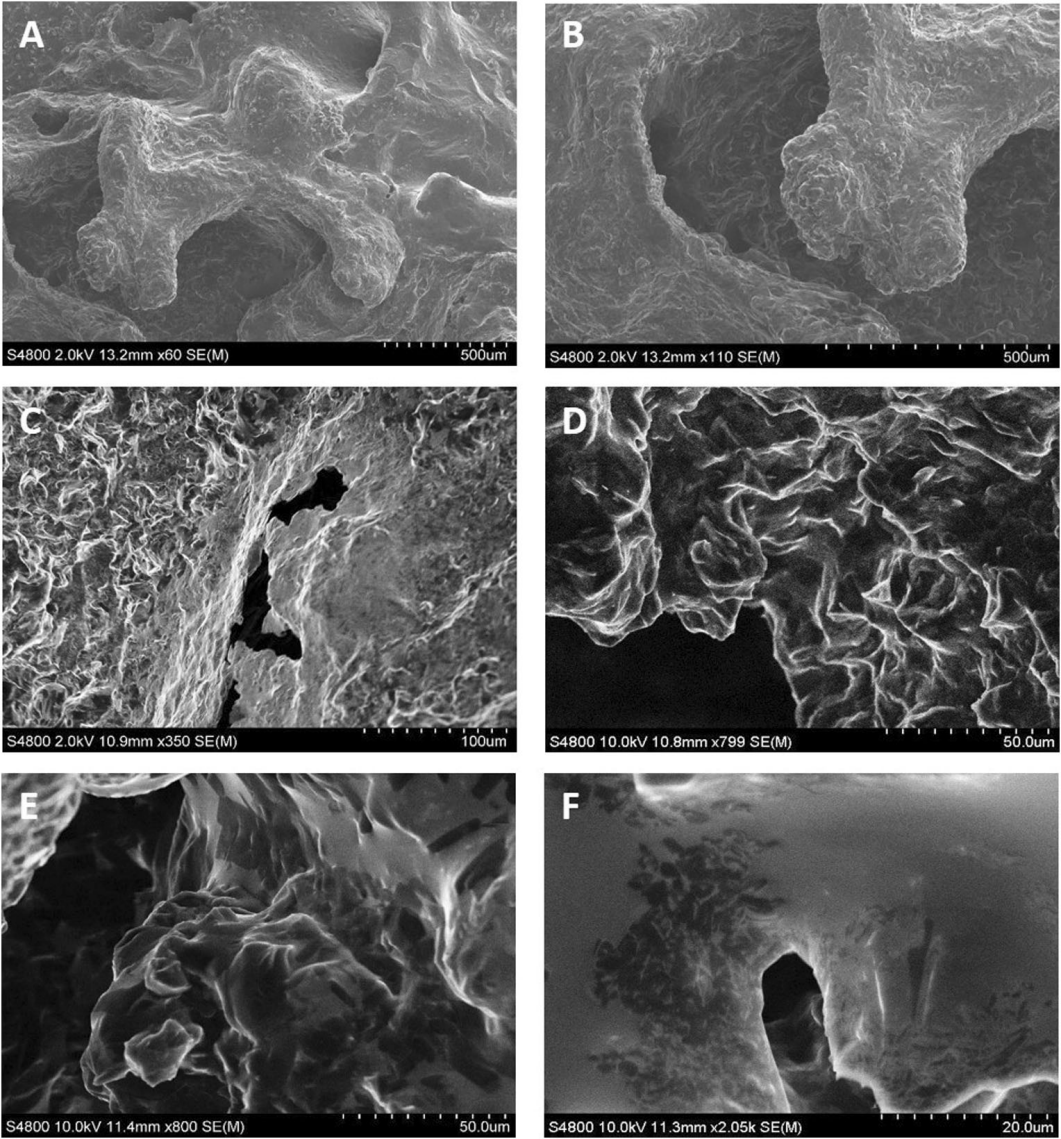
Scanning electron microscope characterization of cubic phase liquid crystal formulations: samples were deposited on a silicon surface, snap frozen in liquid nitrogen to maintain the conformational stability of the liquid crystals and then coated with a 2 nm carbon layer. **a–f** images obtained at 60×, 110×, 350×, 799×, 800× and 2500× respectively, show a crystalline structure typical to cubic phase liquid crystal formulations. Images were acquired with an S-4800 scanning electron microscope (Hitachi) operated at accelerating voltages of 2 or 10 kV

A variety of techniques can be employed for liquid crystal characterization, including small-angle x-ray scattering (SAXS) (Freiberger and Glatter 2006; Goujon et al. 2015; Mendez and Hammouda 2013) and Fourier Transform Infrared Spectroscopy (FTIR) (Koenig et al. 1995; Pongali Sathya Prabu et al. 2011), amongst others, and for an exhaustive analysis of the different techniques used, the reader is referred to the review by An and colleagues (An et al. 2016). In light of the previously published methodology we used to prepare our liquid crystals, we chose scanning electron microscopy (SEM) as a simple and robust approach to liquid crystal characterization. Scanning electron microscopy (Fig. 2) corroborated the findings of other groups working with similar formulations (Nestor et al. 2013; Santamaría et al. 2013). Images obtained at 60×, 110×, 350×, 799×, 800× and 2500× (Fig. 2a–f, respectively) show a crystalline structure typical to cubic phase liquid crystal formulations.

### Liquid crystal emulsions provide a sustained release delivery modality

**Fig. 3:**
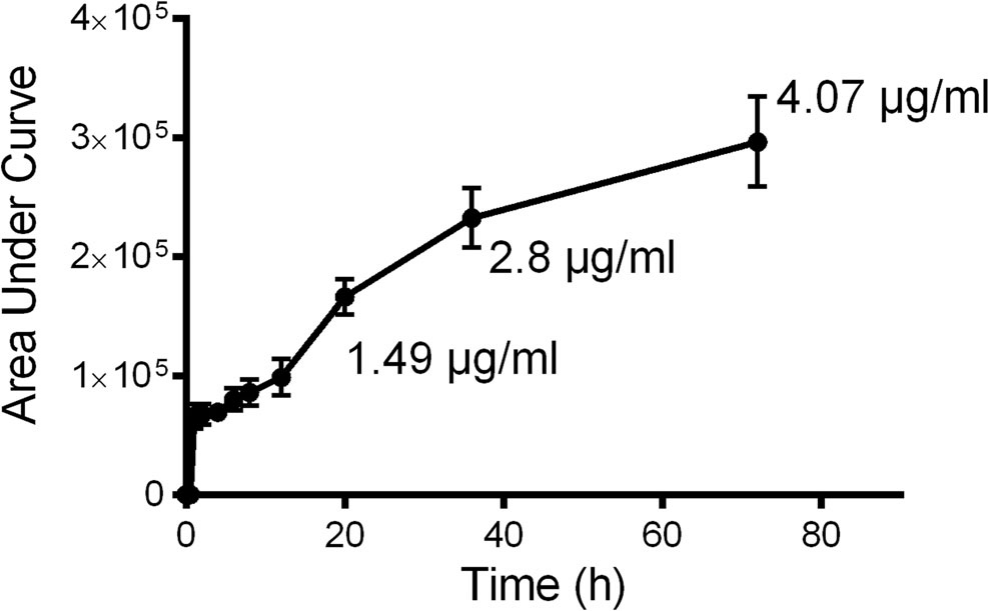
Encapsulated ciprofloxacin displayed sustained release characteristics from cubic phase liquid crystal structures: cubic phase liquid crystal encapsulated ciprofloxacin was sealed in a semi-permeable membrane and placed in a 0.05%TFA solution. Samples were extracted at various time points over a 72-h period. Data shown is mean (SD), *n* = 3. Area under curve (AUC) analysis was performed using GraphPad PRISM 6

After fabrication and characterization of the liquid crystal structures, the rate of release of encapsulated ciprofloxacin was analysed using high pressure liquid chromatography (HPLC). When plotted against a standard curve of ciprofloxacin release (Sup. Fig. 1), area under the curve (AUC) analysis revealed a sustained release profile from cubic phase liquid crystal, peaking at 4.07μg/ml after 72 h (Fig. 3), confirming the suitability of this formulation for sustained, slow-release treatment regimen.

### Liquid crystal formulations have a limited impact on epithelial cell viability

**Fig. 4:**
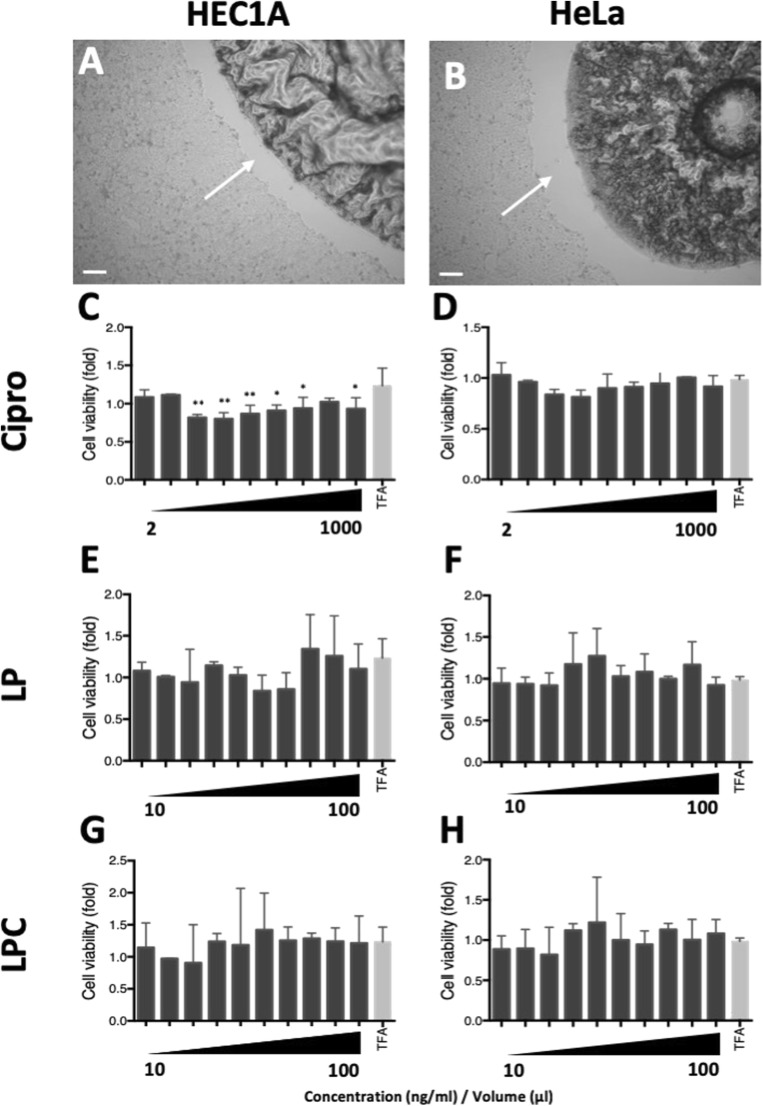
Liquid crystal emulsions have a limited impact on epithelial cell viability: white arrows indicate a corona devoid of cells surrounding the liquid crystal in both cell lines; **a**, **b** Phase-contrast images of the interaction between cubic phase liquid crystal emulsion and HEC1A or HeLa epithelial cells. A corona devoid of cells can be seen around the liquid crystal, but no cell death was observed; **c–h** HEC1A or HeLa epithelial cells were treated with ciprofloxacin (Cipro: 2 ng/ml to 1 μg/ml; C-D), lamellar phase liquid crystal emulsion (LP) (10 μl to 100 μl; E-F) or LP encapsulated ciprofloxacin (LPC) (10 μl to 100 μl ≡ 50 ng/ml to 500 ng/ml of ciprofloxacin; G-H) for 24 h. Scale bars = 500 μm. Data were analysed by ANOVA using Dunnett’s pairwise multiple comparison. Data are presented as mean (SD), *n* = 4. * *p* < 0.05, ** *p* < 0.01 compared to TFA

Before evaluating the efficacy of liquid crystal formulations for the treatment of bacterial infections, we evaluated whether there were any cytotoxic effects of the various formulations on human cells (Fig. 4). HEC1A or HeLa epithelial cells were treated with ciprofloxacin (2 ng/ml to 1 μg/ml), lamellar phase liquid crystal emulsion (LP: 10 μl to 100 μl), LP encapsulated ciprofloxacin (LPC: 10 μl to 100 μl ≡ 50 ng/ml to 500 ng/ml of ciprofloxacin) or the control (TFA in deionised water at the same final concentration as for LPC) for 24 h. Initially, treatments were added directly onto cells. No observable effect was noted following addition of ciprofloxacin to the cell culture (data not shown), but following the addition of liquid crystal emulsions, a corona surrounding the liquid crystal, which was devoid of cells was noted in both HEC1A (Fig. 4a) and HeLa (Fig. 4b) cell cultures. Because of interference between the liquid crystal emulsions and the MTT assay reagents, cell cytotoxicity experiments were performed using Transwell™ support membranes with liquid crystal emulsions held in the Transwell™ and cells in the basal compartment of the plate. Treatment of HEC1A cells with ciprofloxacin resulted in reduced cell counts demonstrating a cytotoxic effect compared to treatment with the control (Fig. 4c). The effect, however, was not dose dependent and interestingly, was not observed in HeLa cells treated with ciprofloxacin (Fig. 4d) or in either HEC1A or HeLa cells once the ciprofloxacin was encapsulated in lamellar phase liquid crystal (LPC, Fig. 4g and h, respectively), suggesting that encapsulated ciprofloxacin is non-toxic to human cells. Treatment of HEC1A (Fig. 4e) or HeLa (Fig. 4f) cells with LP did not affect cell viability compared to control treated cells.

### Encapsulation of ciprofloxacin within a liquid crystal emulsion significantly enhances bacterial killing

Effective and appropriate models for the evaluation of novel therapeutics are an essential tool for drug development. In considering an appropriate model for infection of the female reproductive tract we selected bovine endometrial tissue, which is readily available to us and a suitable model of the human female reproductive tract due to similarities of structure, function and response to bacterial infection (Sheldon et al. 2017; Turner et al. 2012). A common bacterial infection within the bovine female reproductive tract, *E. coli* is one of the most prevalent bacterium associated with uterine disease in cattle. (Sheldon et al. 2009). Thus, we modelled infection of the female reproductive tract using primary bovine endometrial epithelial cells and EVOCs of the bovine endometrium, which have been previously used as effective models of infection, inflammation and for drug development (Borges et al. 2012; Healey et al. 2016; Saut et al. 2014).

**Fig. 5:**
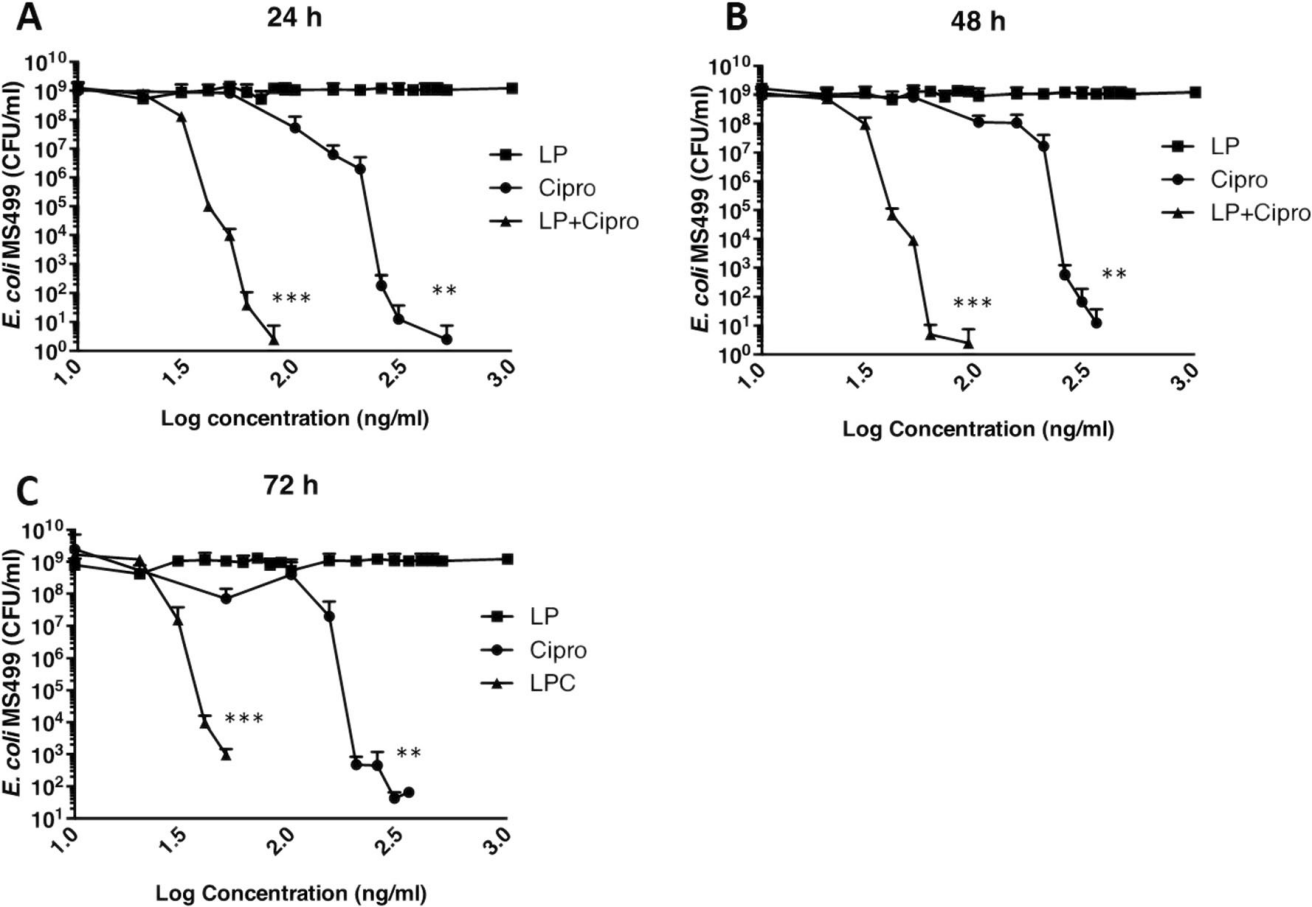
Liquid crystal encapsulation enhances the bacterial toxicity of ciprofloxacin: cultures of *E. coli* (MS499) (at 1 × 10^3^ CFU/ml) were prepared in LB medium and treated with ciprofloxacin (Cipro: 10 ng/ml to 1 μg/ml), lamellar phase liquid crystal emulsion (LP: 10 μl to 100 μl) or LP encapsulated ciprofloxacin (LPC: 10 μl to 100 μl ≡ 50 ng/ml to 500 ng/ml of ciprofloxacin) for 24 h (**a**), 48 h (**b**) or 72 h (**c**). Data were analysed by ANOVA using Dunnett’s pairwise multiple comparison. Data are presented as mean (SD), *n* = 4. ** *p* < 0.01, *** *p* < 0.001 compared to LP

Initial experiments investigated the effect of treatment on bacterial growth alone (Fig. 5). *E. coli* (MS499) was diluted to 1 × 10^3^ colony forming units (CFU)/ml in LB medium for bactericidal experiments and treated with ciprofloxacin (Cipro: 10 ng/ml to 1 μg/ml), lamellar phase liquid crystal emulsion (LP: 10 μl to 100 μl) or LP encapsulated ciprofloxacin (LPC: 10 μl to 100 μl ≡ 50 ng/ml to 500 ng/ml of ciprofloxacin). Bacterial growth in the presence or absence of treatments was monitored for 24 h (Fig. 5a), 48 h (Fig. 5b) or 72 h (Fig. 5c), after which bacterial growth (number of CFU) was determined. At all three time points, treatment with LP had no effect on bacterial growth. Treatment with ciprofloxacin significantly reduced bacterial growth at all time points with no growth observed at ciprofloxacin concentrations above 350 ng/ml. A reduction in bacterial growth was also observed following treatment with LPC, which was significantly greater compared to ciprofloxacin treatment with no growth observed at LPC volumes above 12 μl ≡ 60 ng/ml. To determine the non-inhibitory concentration (NIC) and minimum inhibitory concentration (MIC) for ciprofloxacin (Sup. Fig. 2) and LPC (Sup. Fig. 3) treatments, data was also fitted to a Gompertz model using the method described by Lambert et al (Lambert and Pearson 2000). Table 1 shows the NICs and MICs for the two treatments at 24, 48 and 72 h, demonstrating the enhanced bactericidal effect (lower NIC and lower MIC) of treatment with LPC compared to ciprofloxacin. These data suggested that liquid crystals enhance the effectiveness of antibiotics in the treatment of bacterial diseases.

**Table 1:** Non-inhibitory concentration (NIC) and minimum inhibitory concentration (MIC) of ciprofloxacin and LPC for the treatment of *E.coli* MS499

	NIC (ng/ml)		MIC (ng/ml)	
	Cipro	LPC	Cipro	LPC
24 h	48.96	17.48	97.58	31.26
48 h	52.03	16.59	156.0	36.34
72 h	15.6	19.59	125.1	33.48

Data was fitted to a Gompertz model using the method described by Lambert et al

**Fig. 6:**
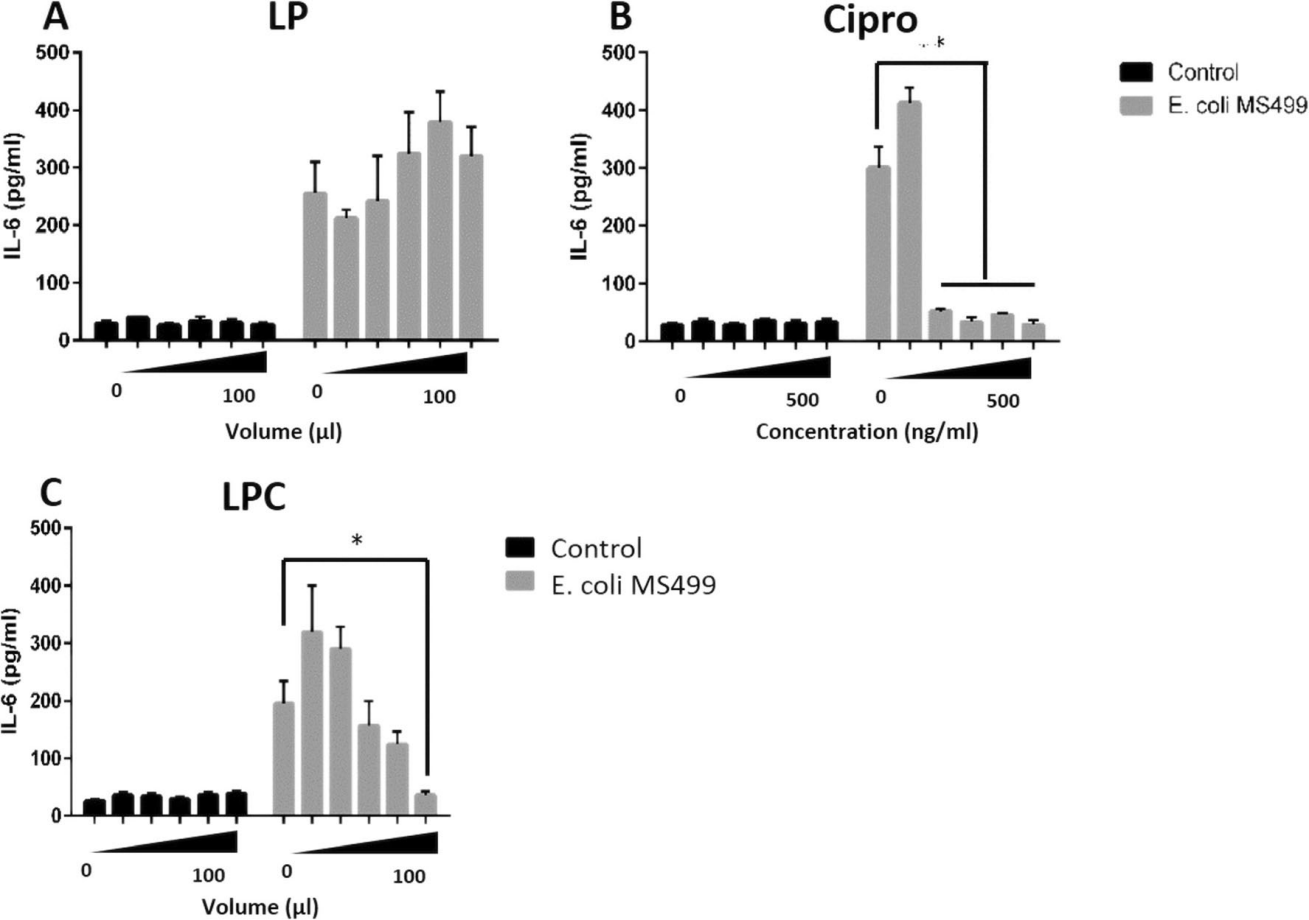
IL-6 secretion by *E. coli* (MS499) infected epithelial cells is reduced following treatment with ciprofloxacin or liquid crystal encapsulated ciprofloxacin: primary bovine epithelial cells were pre-treated with control medium or medium containing ciprofloxacin (Cipro: 100 ng/ml to 500 ng/ml), lamellar phase liquid crystal emulsion (LP: 20 μl to 100 μl) or ciprofloxacin encapsulated liquid crystal emulsion (LPC: 20 μl to 100 μl ≡ 100 ng/ml to 500 ng/ml of ciprofloxacin) for 4 h. Cells were subsequently challenged with control medium or medium containing 1 × 10^3^ CFU/ml *E. coli* (MS499) for 24 h. After challenge, supernatants were collected and stored at −20 °C prior to analysis for IL-6, by ELISA. Data were analysed by ANOVA using Dunnett’s pairwise multiple comparison. Data are presented as mean (SD), *n* = 4. * *p* < 0.05, ** *p* < 0.01

To explore whether reduced bacterial growth would also translate to reduced inflammation, we infected bovine epithelial cells, and EVOCs with *E. coli* (MS499) and monitored inflammatory responses by measuring interleukin (IL)-6 production, which is associated with epithelial driven innate immune responses in the endometrium (Cronin et al. 2012; Healey et al. 2016; Sheldon and Roberts 2010) and responsible for acute and chronic inflammation (Gabay 2006). Epithelial cells or EVOCs, previously treated with ciprofloxacin, LP or LPC for 4 h, were cultured in the presence of control medium or medium containing *E. coli* (MS499) at a concentration of 1 × 10^3^ CFU/ml for 24 h (Fig. 6). Infection of epithelial cells with *E. coli* (MS499) significantly increased the secretion of IL-6 in primary epithelial cell cultures. Treatment with LP (Fig. 6a) did not reduce IL-6 secretion. Conversely, a significant reduction in IL-6 production was noted following treatment with ciprofloxacin concentrations above 200 ng/ml (Fig. 6b) and following treatment with LPC at a volume of 100 μl ≡ 500 ng/ml (Fig. 6c).

**Fig. 7:**
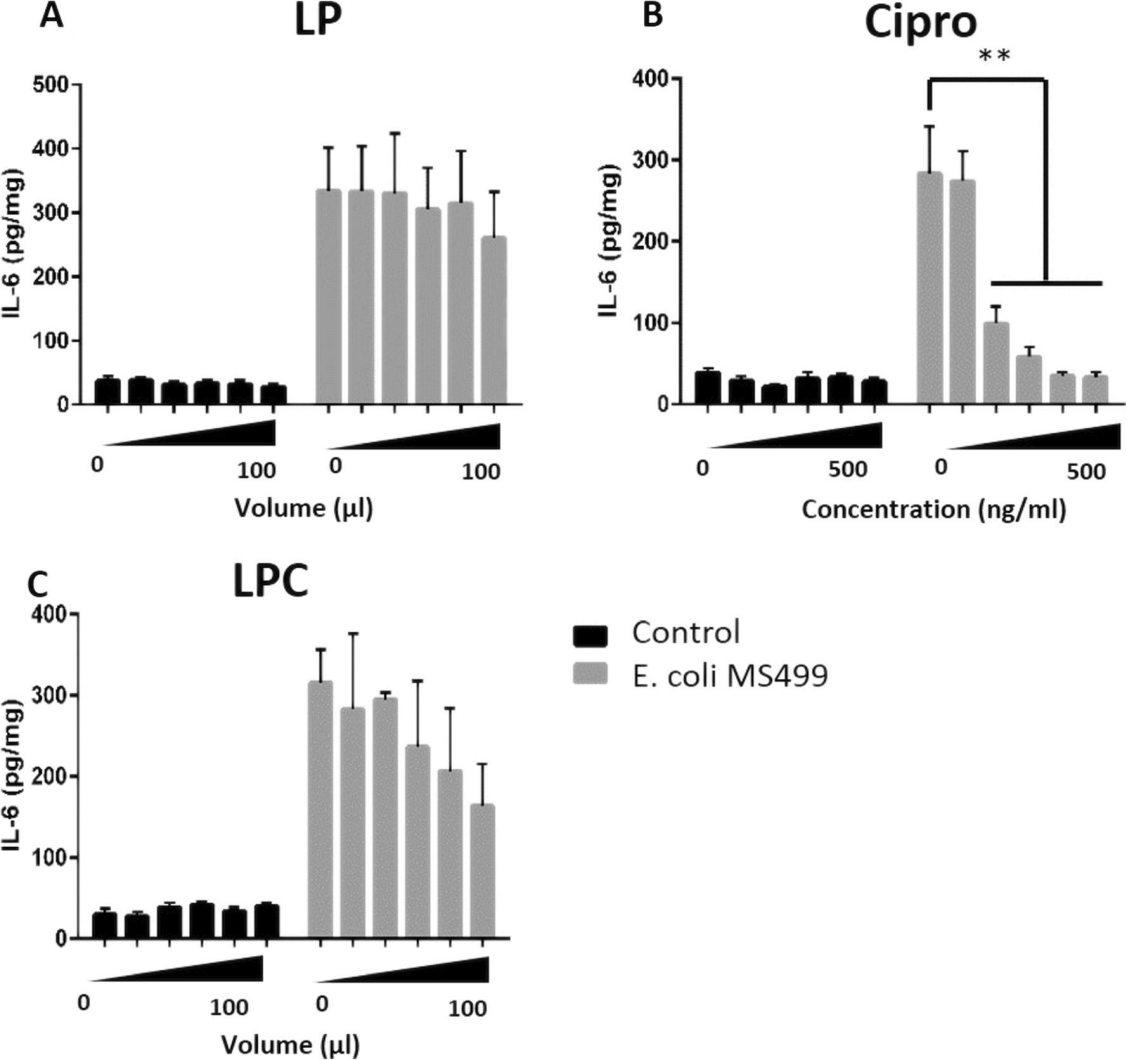
IL-6 secretion by *E. coli* (MS499) infected EVOCs is reduced following treatment with ciprofloxacin or liquid crystal encapsulated ciprofloxacin: EVOCs were pre-treated with control medium or medium containing ciprofloxacin (Cipro: 100 ng/ml to 500 ng/ml), lamellar phase liquid crystal emulsion (LP: 20 μl to 100 μl) or ciprofloxacin encapsulated liquid crystal emulsion (LPC: 20 μl to 100 μl ≡ 100 ng/ml to 500 ng/ml of ciprofloxacin) for 4 h. EVOCs were subsequently challenged with control medium or medium containing 1 × 10^3^ CFU/ml *E. coli* (MS499) for 24 h. After challenge, supernatants were collected and stored at −20 °C prior to analysis of IL-6, by ELISA. EVOC tissues were weighed to enable cytokine concentrations to be adjusted for tissue weight. Data were analysed by ANOVA using Dunnett’s pairwise multiple comparison. Data are presented as mean (SD), *n* = 4. ** *p* < 0.01

As with epithelial cells, infection of EVOCs with *E. coli* (MS499) significantly increased the secretion of IL-6 in primary epithelial cell cultures (Fig. 7). Treatment with LP (Fig. 7a) had no impact on IL-6 secretion and significant reduction in IL-6 production was noted following treatment with ciprofloxacin concentrations above 200 ng/ml (Fig. 7b). For EVOCs treated with LPC, a trend suggesting reduced IL-6 secretion was noted compared to *E. coli* (MS499) infected EVOCs that had not been treated with LPC (Fig. 7c).

## Discussion

In humans, infections of the female reproductive tract are a major socio-economic burden that is associated with significant morbidity and mortality (Horne et al. 2008). Additionally, an increasing prevalence of bacterial resistance over the past two decades means that previously effective antibiotic treatments are being abandoned except in cases where specific sensitivity can be demonstrated through additional testing (Allan-Blitz et al. 2016; Conly and Johnston 2005; So and Shah 2014). There is therefore an urgent need for novel approaches to the treatment of bacterial infections of the female reproductive tract.

Establishing new localised drug delivery vehicles could provide several benefits over conventional therapies that are crucial to microorganism eradication related to bacterial infections. Improved pharmacokinetic and pharmacodynamic profiles reduce the amount of administered drug needed, compared to systemic delivery, to obtain a clinically relevant effect. Improved half-lives and sustained, localised release at concentrations above the bacteria’s minimum inhibitory concentration, when protecting against infection, or above the minimum bactericidal concentration, when treating current infection reduce the incidence of antibiotic resistance (Wu and Grainger 2006). Improved drug efficacy and a reduced need for repeated administrations also reduces costs and enhances patient compliance. Controlled-release platforms such as liquid crystals offer great potential due to their inherent structural characteristics that are ideally suited for encapsulation of antibiotics such as ciprofloxacin. Moreover, the liquid crystal platform presented here affords a slow-release profile, allowing for prolonged treatments avoiding multiple dosing regimens. Furthermore, the platform is minimally invasive and can be applied locally, removing the need for oral or intravenous administration.

Characterization of the liquid crystal formulations demonstrated that they were not toxic to cervical (HeLa) or endometrial (HEC1A) epithelial cells, corroborating previous works (Aldred et al. 2014; Card et al. 2015; Mason et al. 1995). Some cell toxicity was noted in HEC1A cells following treatment with ciprofloxacin, however the effect was not dose dependent suggesting the observed effect might be of limited biological significance. A variety of toxicities associated with ciprofloxacin therapy are known (Amorha et al. 2018; Oyebode et al. 2018; Stahlmann 1990; Stahlmann and Lode 2013) including arthropathogenic potential in animals, which led to the decision to not use ciprofloxacin or other quinolones in children or adolescents. Interestingly, cells treated with ciprofloxacin encapsulated within lamellar phase liquid crystal did not display any toxicity, suggesting the notion that the use of such formulations may be a useful strategy to reduce toxicities associated with current treatment regimen.

In addition to reduced toxicity, ciprofloxacin encapsulation significantly enhanced bacterial cell killing. LPC treatment was five times more effective at killing *E. coli* than free-ciprofloxacin demonstrating the great potential of this formulation for enhancing the efficacy and sustained delivery of antibiotic agents.

Central to the response to bacterial challenge is the detection of pathogen-associated molecular patterns by toll-like receptors (Amjadi et al. 2014). Reproductive tract immune responses to *E. coli* infection require the binding of lipopolysaccharide from *E. coli* to toll-like receptor 4 on the surface of epithelial cells, which then produce IL-6 (Herath et al. 2006; Sheldon and Roberts 2010). The kinetics of IL-6 production reflecting its roles in the early response to infection such as leukocyte recruitment, B lymphocyte development, antibody secretion by plasma cells and the regulation of acute-phase proteins.

Interleukin-6 rapidly accumulates during the first 24 h following exposure to *E. coli* (Sheldon and Roberts 2010), and was attenuated by treatment with free- and encapsulated (LPC) ciprofloxacin. As expected, attenuation was more pronounced in epithelial cells than EVOCs. The purified cell model does not support inflammasome activation and IL1β secretion, which leads to the production of additional pro-inflammatory cytokines, such as more IL-6 (van de Veerdonk et al. 2011), and chemokines such as IL-8 that recruit cells and promote phagocytosis and bacterial clearance (Pétrilli et al. 2007). Although treatment with free-ciprofloxacin appeared more efficacious than treatment with LPC, this is likely due to the concentration of ciprofloxacin present in the culture medium during this 24 h experiment. Whilst the full dose is immediately available following treatment with free-ciprofloxacin, the antibiotic is released slowly over the course of the experiment with LPC. Such a release profile will likely have significant advantages during longer-term infection, whereby the sustained presence of antibiotic will reduce IL-6 production and reduce bacterial load more effectively than the free drug. An expected effect corroborated by the application of liquid crystals for the slow release of drugs with a wide range of molecular weights and water solubilities (Esposito et al. 1996; Geraghty et al. 1996; Nesseem 2001; Nguyen et al. 2011; Nguyen TH et al. 2010; Omray 2013; Wyatt and Dorschel 1992). Myriad factors influence the dynamics of drug release from liquid crystal delivery platforms including temperature, pressure, pH, the molecular weight of the drug, hydrophobicity and salt concentration. Optimization based on the clinical application environment is therefore an important consideration of the drug formulation design process to ensure optimal efficacy.

These data demonstrate that the liquid crystal controlled-release platform presented is effective at delivering antibiotics to treat bacterial infections within the female reproductive tract. Such a platform has potential clinical application supporting a drive towards localised delivery of antibiotic therapies over systemic delivery. Liquid crystal systems have been extensively employed in the clinic as anticancer, anti-inflammatory, analgesic and immunosuppressive agents, amongst others (Chaudhary et al. 2016; Kim et al. 2015; Mei et al. 2018). Indeed, we envisage such a platform being used for topical delivery by the patient for the treatment of lower reproductive tract infections, and/or topical administration by a medical practitioner for upper reproductive tract infections. As well as addressing an important unmet need for efficacious therapies that can reduce negative side effects associated with systemic exposure and offer significant health and economic benefits. Such an approach also provides the potential to increase the efficacy and extend the effective therapeutic life-span of existing antibiotics by reducing the rate of emergence of antibiotic resistance.

## Electronic supplementary material

ESM 1 (JPG 963 kb)

ESM 2 (JPG 54 kb)

ESM 3 (JPG 976 kb)
