# Supplementary figures and images for: Liquid crystal delivery of ciprofloxacin to treat infections of the female reproductive tract
Source: Biomed Microdevices. 2019 Mar 29;21(2):36. doi: 10.1007/s10544-019-0385-x (PMC6439215; doi:10.1007/s10544-019-0385-x)

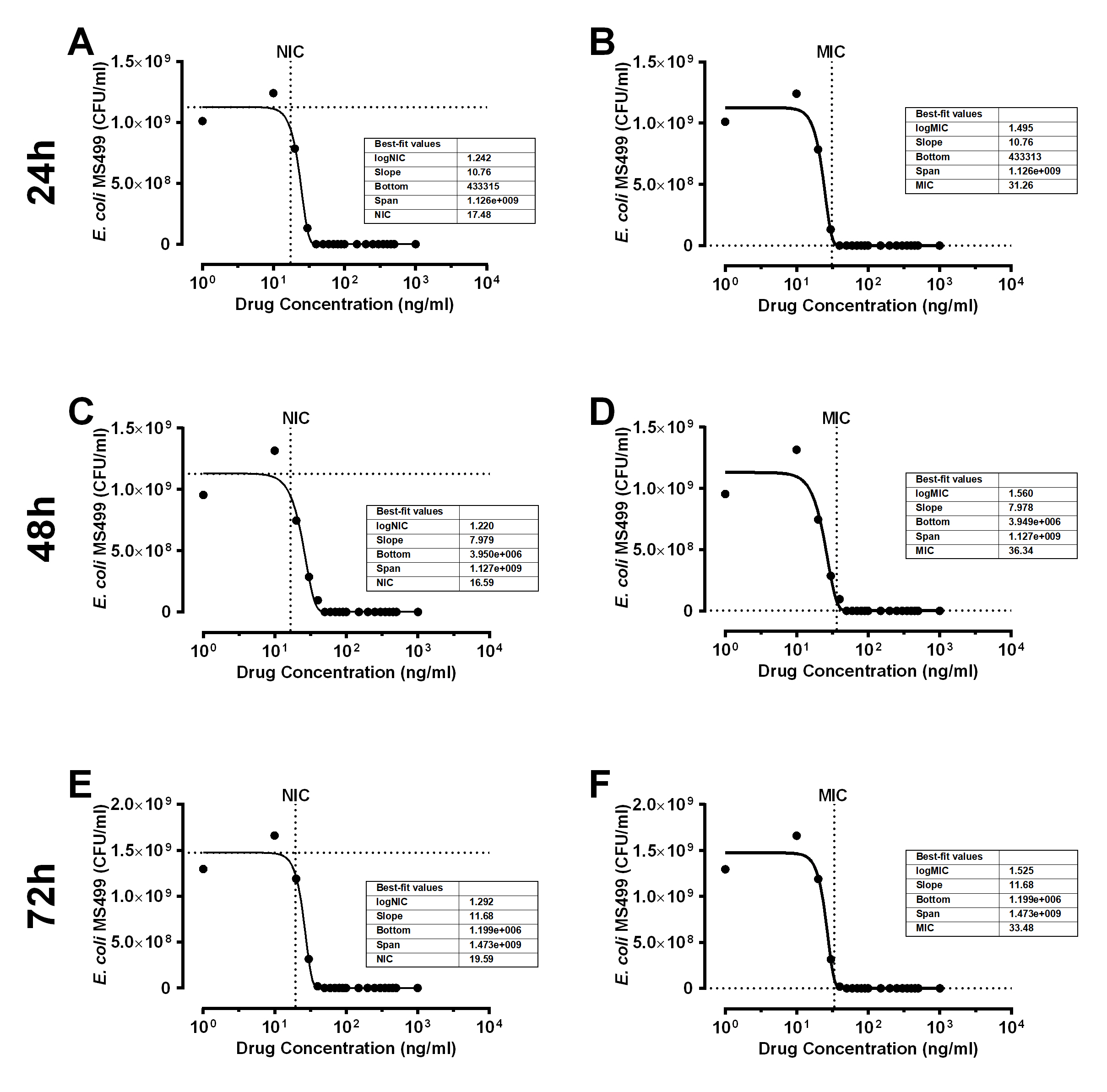

Supplement: Supplementary file 1 — (JPG 963 kb) [file 10544_2019_385_MOESM3_ESM.jpg]

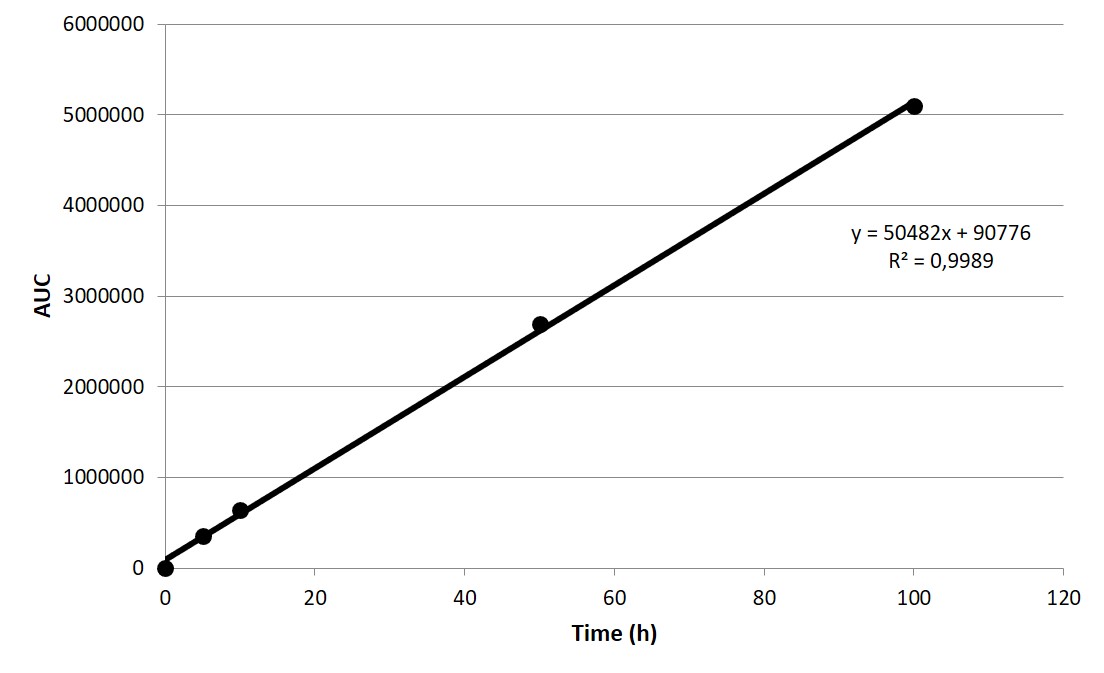

Supplement: Supplementary file 2 — (JPG 54 kb) [file 10544_2019_385_MOESM1_ESM.jpg]

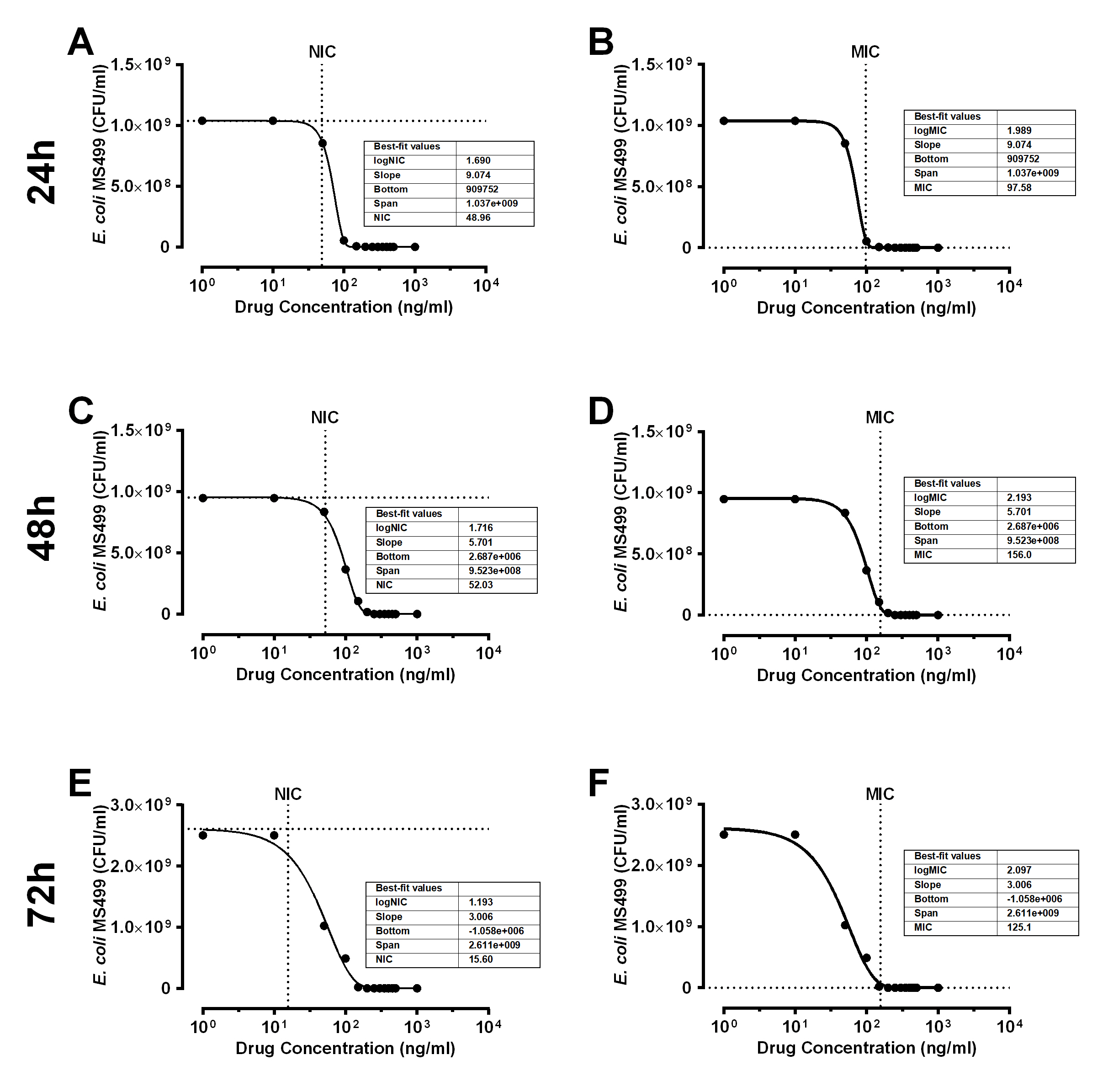

Supplement: Supplementary file 3 — (JPG 976 kb) [file 10544_2019_385_MOESM2_ESM.jpg]
